# Tuberculosis and How to Combat It

**Published:** 1915-09

**Authors:** 


### TUBERCULOSIS AND HOW TO COMBAT IT.

The following simple rules for the prevention of tuberculosis in school children have been elaborated by Dr. S. A. Knopf, the famous New York physician, who has contributed so much to the study of the disease:

Every child and grown person can help to fight consumption. School children can help by obeying the following rules:

Do not spit except in a spittoon, a piece of cloth or a handkerchief used for that purpose alone. On your return home have the cloth burned by your mother or the handkerchief put in water until ready for the wash.

Never spit on a slate, floor, playground or sidewalk.

Do not put your fingers into your mouth.

Do not pick your nose or wipe it on your hand or sleeve.

Do not wet your fingers in your mouth when turning the leaves of books.

Do not put pencils in your mouth or wet them with your lips.

Do not hold money in your mouth.

Do not put pins in your mouth.

Do not put anything in your mouth except food and drink.

Do not swap apple cores, candy, chewing gum, half-eaten food, whistles, bean-blowers or anything that is put in the mouth.

Peel or wash your fruit before eating it.

Never sneeze or cough in a person's face. Turn your face to one side or hold a handkerchief before your mouth.

Keep your face, hands and finger nails clean. Wash your hands with soap and water before each meal.

When you don't feel well, have cut yourself or have been hurt by others do not be afraid to report to the teacher.

Keep yourself just as clean at home as you do at school.

Clean your teeth with toothbrush and water, if possible, after each meal; but at least on getting up in the morning and on going to bed at night.

### When the Physician Should Be Consulted.

In a recent issue of the *Journal of the Outdoor Life* the following symptoms are given, any one of which, it is pointed out, should lead to a visit to a physician for a thorough examination of the lungs.

1. A cough lasting a month, excepting whooping cough.
2. Poor appetite (especially in the morning) and indigestion, loss of weight and strength, and pallor (generally "run down").
3. Hoarseness, lasting several weeks.
4. Spitting, especially in the morning.
5. Night sweats.
6. Spitting blood.
7. Fever in the afternoon, shown by flushed face and tired feeling.

Any, several or all of these symptoms coming after a severe cold, grip, bronchitis, whooping cough, measles, typhoid fever or any other acute disease may indicate tuberculosis.

Two or three or more examinations should be made. The germs may not be found the first time the sputum is examined, and, indeed, they may not be found at all until the disease is far advanced. Accordingly no reliance should be placed on a negative sputum examination, if other symptoms are present. A thorough physical examination should be had, and perhaps several of them. Tuberculosis in its incipient stage is a very difficult disease to diagnose even in a physical examination.

